# A Facile yet Versatile Strategy to Construct Liquid Hybrid Energy‐Saving Windows for Strong Solar Modulation

**DOI:** 10.1002/advs.202206044

**Published:** 2023-01-20

**Authors:** Jichang Li, Pengyu Gu, Hongyu Pan, Zhiyuan Qiao, Jianfeng Wang, Yanxia Cao, Wanjie Wang, Yanyu Yang

**Affiliations:** ^1^ School of Materials Science and Engineering Zhengzhou University Henan 450001 P. R. China

**Keywords:** energy‐saving, liquid smart windows, solar modulation, synergetic mechanisms, tunable transition temperature

## Abstract

Smart windows with light management and indoor solar heating modulation capacities are of paramount importance for building energy conservation. Thermochromic poly(*N*‐isopropylacrylamide) (PNIPAm) hydrogel smart windows exhibit advantages of the relatively suitable transition temperature of 32 °C, high cost‐effective and automatic passive sunlight regulation, but sustain slow response rate and unsatisfactory solar modulation efficiency. Herein, a strategy of one‐step copolymerization of NIPAm and different olefine acids (OA) using reverse atom transfer radical polymerization method is developed to fabricate various chain/microparticle hybrids (CMH) for liquid energy‐saving windows. Synergetic mechanisms of thermal‐induced dissolution and aggregation of linear polymer chains integrated with water capture and release of microgel particles contribute to tunable light‐scattering behaviors and adaptive solar modulation. Without any post‐treatment, the as‐prepared poly(*N*‐isopropylacrylamide‐*co*‐acrylic acid) (P(NIPAm‐*co*‐AA))‐based CMH suspension is injected into sandwich glass to construct energy‐saving windows, which exhibits appreciated near‐room‐temperature transition (26.7 °C), rapid response (5 s), extraordinary luminous transmittance (91.5%), and solar modulation efficiency (85.8%), resulting in a substantial decline of indoor temperature of 24.5 °C in simulation experiment. Combining the versatile strategy with flexible adjustment on transition temperature, multifarious P(NIPAm‐*co*‐OA)‐based CMH windows with eminent light management capacity are obtained. This work will powerfully promote the development and renovation of energy‐efficient windows.

## Graphical abstract

Diversified liquid hybrid smart windows based on synergetic mechanisms of thermal‐induced dissolution and aggregation of linear chains and water capture and release of microgel particles are developed via facile copolymerization of *N*‐isopropylacrylamide and various olefine acids. The windows possess easy large‐scale production, near‐room‐temperature transition, extraordinary light management, and indoor temperature regulation capacity, holding promises in commercial energy‐saving building materials.

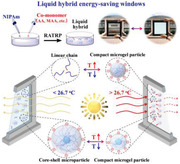

## 1 Introduction

Achieving carbon neutrality in the mid‐21st century is the world's most urgent mission, which has induced extensive forcible policy measures to boost energy conservation and emission reduction.^[^ ^1^ ^]^ Buildings account for almost 40% of energy consumption in the world, half of which is utilized for heating, ventilation, and air conditioning.^[^ ^2^ ^]^ As the least energy efficient part of building envelope, windows play indelible role in heat gain induced by window‐directed solar energy in summer and heat loss by thermal conduction in winter.^[^ ^3^ ^]^ Pursuing smart windows that dynamically regulate the indoor solar heating via enhancing or reducing the sunlight irradiation is of pivotal significance for building energy saving and carbon emission reduction. According to the type of chromogenic technologies, current smart windows can be categorized as electrochromism,^[^ ^4^ ^]^ photochromism,^[^ ^5^ ^]^ mechanochromism,^[^ ^6^ ^]^ and thermochromism.^[^ ^7^ ^]^ Without additional electric supply or complicated fabrication process, the thermochromic windows exhibit remarkable advantages of automatic passive controllability, zero energy input, high cost‐effective, and feasible implementation.^[^ ^8^ ^]^ Depending on the environmental temperature, the thermochromic windows can spontaneously adjust the solar transparent or opaque state to meet the switching demands in cold and hot weather, being regarded as great competitive energy‐saving windows.

Vanadium dioxide (VO_2_) and hydrogel are the two most attractive materials for thermochromic windows. VO_2_ can switch infrared‐transparent semiconducting state into infrared‐reflective metallic state for achieving near‐infrared (NIR) regulation.^[^ ^9^ ^]^ However, the high phase transition temperature (*T*_c_) of 68.0 °C, as well as the poor stability, severally restrict the practical application of VO_2_ based smart windows.^[^ ^10^ ^]^ Introduction of dopants (tungsten, niobium, and magnesium) into VO_2_ based windows can decrease the transition temperature by 10.8 °C, while impeding the luminous transmittance (*T*_lum_) and solar transmittance modulation (Δ*T*_solar_).^[^ ^10^, ^11^ ^]^ Poly(*N*‐isopropylacrylamide) (PNIPAm) hydrogels, with a relatively lower transition temperature of 32 °C, can reversibly capture and release water upon phase transition, resulting in tuning optical property and greater prospect of building energy conservation than VO_2_.^[^ ^12^ ^]^ However, their weak modulating capacity in NIR region that contributes about 53% of solar energy inevitably leads to low energy‐saving efficiency.^[^ ^13^ ^]^ Although incorporating VO_2_,^[^ ^14^ ^]^ Ag nanoparticles,^[^ ^15^ ^]^ antimony tin oxide (ATO),^[^ ^16^ ^]^ and cesium tungsten bronze (Cs*_x_* WO_3_)^[^ ^17^ ^]^ into PNIPAm hydrogels can improve the NIR regulation ability, the luminous transmittance was substantially impaired. Recently, the developed liquid PNIPAm‐based windows revealed shape independence, scalability, and volume stability,^[^ ^8^, ^18^ ^]^ compared to conventional hydrogel windows. By controlling the particle size and internal structure of microgel using a continuous feeding method, broadband spectral management of smart windows can be realized with remarkable solar modulation efficiency of 81.3%.^[^ ^18^ ^]^ Nevertheless, the design of liquid high‐performance PNIPAm‐based smart windows was restricted in specific systems. Additionally, nearly all liquid PNIPAm‐based smart window materials require tedious concentration and extra surfactant to enhance the solar modulation and long‐term stability, respectively.^[^ ^8^, ^13^, ^18^, ^19^ ^]^ Furthermore, the transition temperature slightly above room temperature and slow response rate is also urgent problems to be addressed for practical application. Hence, exploiting a versatile strategy to prepare thermochromic smart windows integrated near‐room‐temperature transition, rapid response with strong solar modulation capacity are great worth for building energy conservation.

Herein, we proposed a hitherto unexplored strategy of one‐step copolymerization of *N*‐isopropylacrylamide (NIPAm) and acrylic acid (AA) using reverse atom transfer radical polymerization (RATRP) method to prepare chain/microparticle hybrid (CMH) for highly energy‐saving smart windows. The liquid CMH suspension consisted of distinctive linear P(NIPAm‐*co*‐AA) chains and Cu_3_Cit_2_@P(NIPAm‐*co*‐AA) core–shell microgel particles. The synergetic mechanisms of dissolution‐aggregation conversion of polymer chains and the water capture‐release transformation of microgel particles enabled the tunable light scattering behavior and prominent solar modulation capacity. Without any post‐treatment, the as‐prepared CMH suspension was poured into sandwich glass to fabricate thermochromic smart window, which demonstrated appreciated transition temperature (26.7 °C), extraordinary luminous transmittance (91.5%) and eminent light regulation capacity in NIR region (78.7%) and full solar region (85.8%). During multiply heating‐cooling cycles, the smart window revealed superiorly stable solar modulation property due to the strong electrostatic repulsion among microgel particles. Remarkably, a substantial decline in indoor temperature of 24.5 °C was achieved by utilizing the CMH smart window. Simple varying the pH value of CMH suspension can achieve flexible regulation of transition temperature in a broad range from 26.7 to 82.0 °C. Significantly, the strategy can be generalized to various olefine acid (OA) co‐monomers (acrylic acid, methacrylic acid, 3‐butenoic acid, and 4‐pentenoic acid) for yielding diversified energy‐saving CMH smart windows. The versatile strategy of P(NIPAm‐*co*‐OA)‐based CMH smart windows realized feasible one‐step construction, easy large‐scale production, near‐room‐temperature transition with overwhelming luminous transmittance and solar modulation capacity.

## 2 Results and Discussion

**Figure 1:**
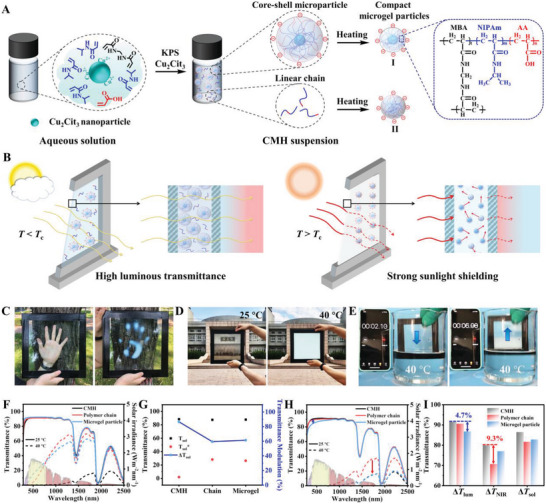
A) Schematic preparation of high‐concentration CMH suspension via RATRP method and the structural variation upon heating. B) Solar modulation mechanism of CMH SW. C,D) Optical variation of CMH SWs upon hand touching and heating treatment. E) Rapid response of CMH SW at 40 °C. F) Transmittance spectra of various SWs with CMH supension, separated polymer solution, and separated microparticle suspension as interlayers at 25 and 40 °C. The inset was the solar irradiance spectrum. G) The corresponding solar transmittances (*T*_sol_) and solar transmittance modulations (Δ*T*_sol_). H) Transmittance spectra of various SWs with identical concentration of liquid interlayer materials at 25 and 40 °C. G) The corresponding light transmittances (*T*_lum_, *T*_NIR_, and *T*_sol_) and light transmittance modulations (Δ*T*_lum_, Δ*T*_NIR_, and Δ*T*_sol_). The interlayer thickness of SWs was 280 µm.

As illustrated in **Figure** **1**A, the thermochromic P(NIPAm‐*co*‐AA)‐based CMH suspension was prepared via simple one‐step copolymerization of NIPAm and AA via controlled RATRP method. In detail, high‐concentration monomers of NIPAm and AA with molar ratio of 5:1, *N*,*N*‐methylenebis(acrylamide) (MBA, crosslinker), potassium persulphate (KPS, initiator) and copper citrate (Cu_3_Cit_2_, copper compound with high oxidation state) were dissolved in DI water, wherein massive Cu_3_Cit_2_ nanoparticles were dispersed in the solution due to low solubility. Then, the mixture was polymerized at 70 °C for 3 h to simultaneously obtain high‐concentration P(NIPAm‐*co*‐AA) chain and microparticle in the suspenson. During the polymerization process, divalent copper ions can capture the radicals to yield dormant species, and further maintain the balance of active species between dormant species, remaining slow polymerization rate and effectively avoiding the formation of bulk hydrogel.^[^ ^20^ ^]^ The complexation of copper ions with NIPAm and AA also benefited to lower the polymerization rate, but also made the insoluble polymer aggregate toward the suspending Cu_3_Cit_2_ particles into large‐size core–shell microgel particles. The electronegativity of carboxyl groups availed the loose structure of core–shell microgel particles and consequently reduced the extinction coefficient and refractive index, achieving highly transparent core–shell microgel particles.^[^ ^21^ ^]^ Compared to NIPAm and AA with similar reactivity ratios, the crosslinker MBA possessed higher reactivity ratio, leading to rapid consumption of crosslinker at the early stage of polymerization.^[^ ^22^ ^]^ At the later stage of polymerization, the long decomposition course of initiator KPS as well as the higher molar quantity of KPS than copper ions contributed to concurrent linear copolymerization of NIPAm and AA. After the polymerization was completed, the liquid CMH suspension contained high‐concentration linear P(NIPAm‐*co*‐AA) chains (3.3 wt%) and core–shell microgel particles (3.5 wt%). Beyond transition temperature, the original swollen core–shell microgel particles (I) squeezed internal water into collapsed microgel particles (I) with smaller diameter, meanwhile the P(NIPAm‐*co*‐AA) chain switched random‐coil conformation into tightly packed globular conformation^[^ ^23^ ^]^ for yielding newly‐formed compact microgel particles (II). Without any post‐treatment, the as‐prepared CMH suspension was injected in sandwich glass to construct CMH smart windows (SWs) for achieving adaptive solar modulation. The feasible liquid phase preparation of CMH SWs permitted prominent advantages of high cost‐effective and large‐scale production, manifesting the huge potential in industrialization and practical applications.

The core–shell structure of microgel particles (I) was obviously observed by super depth of field microscope (SDFM) using lateral light source. As shown in Figure S1, Supporting Information, the high‐concentration water‐rich microgel particles (I) were dispersed in water, wherein the inner cores were blue Cu_3_Cit_2_ nanoparticles (≈150 nm, Figure S2, Supporting Information). Additionally, after adding NaOH solution, the transparent CMH suspension turned into stable reddish‐brown suspension without any precipitate, while the pure Cu_3_Cit_2_ suspension yielded reddish‐brown precipitate, powerfully verified that the Cu_3_Cit_2_ nanoparticles were wrapped in the microgel particles (I) (Figure S3, Supporting Information). The core–shell structure of microgel particles (I) was in favor of large particle size and loose internal structure and contributed to high light transmittance below transition temperature. Thus, the high‐concentration CMH suspension was highly transparent at RT, compared to the highly turbid PNIPAm microgel suspension (1 wt%) prepared via classical method^[^ ^24^ ^]^ (Figure S4, Supporting Information).

The synergetic mechanisms of thermal‐induced dissolution and aggregation behavior of linear polymer chains and the water capture and release behavior of microgel particles contributed to tuning scattering behavior and dynamic solar modulation. As shown in Figure 1B, at low temperature (<*T*_c_) in cloudy condition, the random coil state of P(NIPAm‐*co*‐AA) chains and highly swollen state of microgel particles (I) led to insignificant interfaces and index matching between CMH (1.34) and water (1.33), allowing sunlight to easily pass through the window for warming up indoor environment (Figure S5A, Supporting Information). On the contrary, at high temperature (>*T*_c_) in sunny condition, the P(NIPAm‐*co*‐AA) chains underwent dissolution‐to‐aggregation transition to yield newly‐formed compact microgel particles (II), meanwhile the core–shell microgel particles (I) underwent hydrophilic‐to‐hydrophobic transition and sharp volume shrinkage (Figure S5B and Video S1, Supporting Information), inducing strong optical contrast between microgel particles with water and resultant strong light scattering. The strong light scattering can effectively reduce the radiant heat arriving indoors, achieving passive cooling and energy conservation in summer.

A large CMH SW (30 × 30 cm^2^) was easily fabricated and displayed high transparency to clearly observe the near tree and distant building at RT (Figure 1C,D). Upon hand touching or heating treatment at 40 °C, the CMH SW quickly underwent phase transition and turned solar opaque to lose sight of views, indicating the switchable optical property. In contrast to core–shell microgel phase transition involving the hydrophilic‐to‐hydrophobic transition of crosslinked network and water capture and release, the dissolution‐to‐aggregation transition of linear P(NIPAm‐*co*‐AA) chains involved only hydrophilic‐to‐hydrophobic transition of molecular chains, contributing to fast coil‐globular conformation transition and a prompt response to environmental temperature (Figure S6, Supporting Information). The quickly reversible coil‐globular conformation transition of linear P(NIPAm‐*co*‐AA) chain enables the CMH SW to demonstrate rapid response and recovery in just 5 s (Figure 1E; Figure S7 and Video S2, Supporting Information). Moreover, the CMH suspension was able to remain stable volume during heating‐cooling cycles (Figure S8, Supporting Information). The fast response to environmental temperature and volume stability of liquid CMH SW was beneficial to enhance the energy conservation and utility value.

In order to confirm the above‐mentioned synergetic mechanisms, the linear chain (3.3 wt%) and microgel particle (3.5 wt%) were separated via centrifugalization and further utilized to fabricate SWs to evaluate individual solar regulation capacity. As illustrated in Figure 1F,G, the SWs prepared from polymer chain and microgel particle displayed inferior solar modulation efficiencies of 59.3% and 61.8%, respectively. However, the CMH SW revealed a ultrahigh solar modulation value of 85.8%. The striking comparison indicated that the excellent solar regulation capacity of CMH SW originated from the synergetic effect of polymer chain and microgel particle. Notably, the aqueous solution phase of PNIPAm‐based materials for energy‐saving window grounded on dissolution‐aggregation transition was reported for the first time. Then, various SWs with identical concentration (6.8 wt%) of liquid interlayer materials (CMH, linear chain, and microgel particle) were also fabricated to evaluate the light management ability. As illustrated in Figure 1H,I, compared to weak NIR regulation of polymer chain SW and the decreased luminous transmission of microgel SW at RT, the CMH SW displayed extraordinary luminous transmission (91.5%) and eminent light regulation capacity in NIR region (78.7%) and full solar region (85.8%). Consequently, the CMH SW based on synergetic mechanisms displayed eminent advantages in broadband light management.

Compared with solid hydrogel smart windows, the liquid phase smart windows integrate easy fabrication with volume stability, exhibiting superior probability in commercialization. In this work, both divalent copper ions and AA played indispensable roles in the formation of liquid high‐concentration CMH suspension rather than bulk hydrogel. The initiating system consisted of bivalent copper ions and KPS can realize the reversible balance of radical active species and dormant species, reducing the polymerization rate and achieving controlled polymerization.^[^ ^20^ ^]^ Moreover, the complexation between AA and copper ions, together with the acid environment provided by AA, also worked in tandem to lower the polymerization rate.^[^ ^25^ ^]^ Besides, the electronegativity of carboxyl groups rendered the electrostatic repulsion among microgel particles, avoiding aggregation and linkage in the polymerization process. Hence, the polymerization systems without divalent copper salt or AA generated opaque bulk hydrogels (Figure S9, Supporting Information), whereas all of the RATRP polymerization systems containing various copper salts (CuCl_2_, CuSO_4_ or Cu_3_Cit_2_) and AA yielded liquid CMH suspensions (Figure S10, Supporting Information). Notably, only the microgel particles (I) prepared with low solubility of Cu_3_Cit_2_ demonstrated core–shell structure and consequently larger particles size and looser internal structure compared with other systems, resulting in superior solar transmittance of 87.8% at RT. As the temperature increased to 40 °C, all SWs underwent phase transition to block intense solar irradiance. The Δ*T*_sol_ of SW‐Cl, SW‐Sul, and SW‐Cit were 61.9%, 75.3%, and 85.8%, respectively. On the basis of the extraordinary solar transmittance modulation, the CMH SW‐Cit was used for the following investigation. Moreover, the molar ratio of NIPAm to AA affected the internal interaction (electrostatic repulsion and hydrogen bonding) and hydration degree of CMH, as well as the transparency of CMH suspension at RT. As illustrated in Table S1 and Figure S11, Supporting Information, as the *n*_(NIPAm)_:*n*_(AA)_ was 10:1, the internal weak electrostatic repulsion led to a relatively dense structure of microgel particles (I), resulting in a turbid CMH suspension at RT. When the *n*_(NIPAm)_:*n*_(AA)_ was 2:1, the low pH value significantly enhanced the molecular hydrogen bonding within CMH, resulting in a low transition temperature below RT. The CMH suspension was solar opaque at RT, but turned into highly transparent at 5 °C (Figure S11C,C', Supporting Information). In comparison, as the *n*_(NIPAm)_:*n*_(AA)_ was 5:1, the CMH suspension was highly transparent at RT due to the strong internal electrostatic repulsion and loose structure of microgel particles (I), allowing high light transmission of the assembled window.

A near‐room‐temperature transition was highly appreciated for thermochromic energy‐saving windows. As the temperature increased, the hydrogen bonding within chains and microgel particles enhanced, and the hydrogen bonding between chains and microgel particles with water molecules reduced, leading to rapid dehydration and phase transition. Differential scanning calorimetry (DSC) was utilized to determine the transition temperature. As illustrated in Figure S12, Supporting Information, the CMH suspension demonstrated a lower transition temperature of 26.7 °C, compared with 31.8 °C of PNIPAm hydrogel. Meanwhile, the dynamic transmittance measurement declared that the transition temperature of CMH suspension was 26.9 °C, which was very close to the result of DSC measurement (Figure S13, Supporting Information). The reduction of transition temperature was associated with the enhanced molecular hydrogen bonding induced by the additional polar carboxyl groups together with the provided acid environment, as well as the higher sensitivity of linear polymer chains to temperature compared to crosslinked microgel or hydrogel. Impressively, the transition temperature of CMH suspension was close to the optimum indoor temperature for human beings, which was advantageous to smart windows in practical applications.

**Figure 2:**
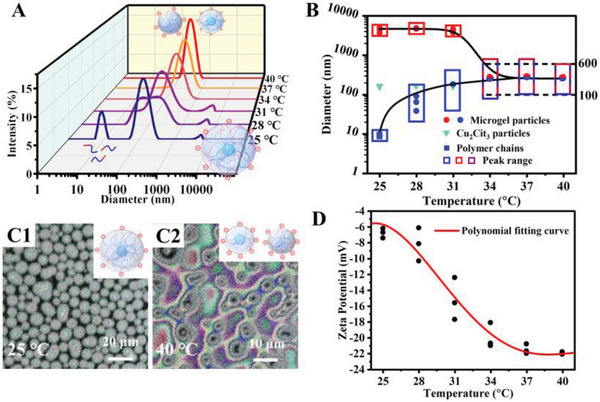
A) DLS curves of CMH suspension at different temperatures. B) Hydrodynamic diameters evolution of microgel particles in response to raised temperature. C) SDFM images of CMH suspension at 25 and 40 °C. D) Zeta potentials of microgel particles at different temperatures.

The hydrodynamic diameter evolution of CMH in response to raised temperature was measured via dynamic light scattering (DLS) in **Figure** **2**A. At 25 °C, there were three peaks (≈10 nm, 150 nm, and 5 µm) in the DLS curve of CMH suspension, which were attributed to the linear P(NIPAm‐*co*‐AA) chains, central Cu_3_Cit_2_ particles and core–shell microgel particles (I), respectively. As the temperature increased to 28 °C, enhanced intermolecular hydrogen bonding and impaired interaction between P(NIPAm‐*co*‐AA) chains with water significantly promote the physical aggregation of linear chains, generating microgel particles (II) with a broad peak of 20–200 nm. With the further increase in temperature, the newly‐formed microgel particles (II) became larger. In the meantime, the internal hydrogen interaction of microgel particles (I) enhanced drastically to release inner water, resulting in substantial volume shrink. Finally, two peaks in DLS curves evolved into one peak at 34 °C and further narrowed at 40 °C. The detailed size variation of microgel particles was illustrated in Figure 2B. The red and blue rectangles represented the original microgel particles (I) and new‐formed microgel particles (II), respectively, and the top and bottom lines of rectangle represented the peak range of microgel particles. Finally, the red and blue rectangles merged into a rectangle, and the corresponding particle size range was 100–600 nm. The dimensionless size parameter *α* (2*π*r/*λ*) comparable with 1 in the visible and almost NIR region, contributing to strong Mie scattering in almost full solar spectrum and broadband light management ability.^[^ ^18^ ^]^ Additionally, optical images visually demonstrated the morphology changes of microgel particles and their tuning light‐scattering behavior. As shown in Figure 2C, at 25 °C, the highly swollen core–shell microgel particles (I) exhibited homogeneously low mismatch of refractive index with water. At 40 °C, the collapsed microgel particles (black dots in Figure 2C2) generated remarkable different refractive index with water and consequently strong scattering and colorful rims.^[^ ^26^ ^]^ The above results clearly manifested the great potential of CMH suspension for powerful solar modulation.

The geometric morphology and dimensional variation in SDFM images and hydrodynamic diameter evolution in DLS curves clearly illustrated the hydrophilic‐to‐hydrophobic transition and water capture‐to‐release of the core–shell microgel particles (I). To obviously illustrate the dissolution‐to‐aggregation transition of linear P(NIPAm‐*co*‐AA) chains, the linear P(NIPAm‐*co*‐AA) chains were separated from the CMH suspension for DLS measurement to demonstrate the changes in particle size. As illustrated in Figure S14, Supporting Information, the separated P(NIPAm‐*co*‐AA) solution only displayed a peak of 10 nm at 25 °C. At 40 °C, the original peak disappeared, and a new peak of 100–500 nm appeared, meaning the formation of microgel particles (II) from P(NIPAm‐*co*‐AA) chains. The hydrodynamic diameter evolution of separated P(NIPAm‐*co*‐AA) chains clearly proved the phase transition from random‐coil conformation to globular conformation.

Zeta potential measurement was employed to test the surface charge of microgel particles during heating process and evaluate their dispersed stability.^[^ ^27^ ^]^ With the increase in temperature, the zeta potential revealed a smoothly declining trend (Figure 2D), which was quite different from the dramatic drop in the previous literatures.^[^ ^8b^ ^]^ The zeta potential gradually decreased from −6 mV at 25 °C to −20 mV at 34 °C, then stabilized at −23 mV at 37 °C. The downward tendency was attributed to the increased ionization degree of carboxyl groups and the elevated surface negative charge density along with the volume shrinkage of microgel particles (I). The enhanced electrostatic repulsion among microgel particles was conducive to maintain their stable dispersion during multiply heating process. As shown in Figure S15, Supporting Information, the CMH suspension remained stable at 40 °C for 6 consecutive days. Despite the slight stratification on the seventh day, the suspension recovered remarkable transparency when replaced at 25 °C.

**Figure 3:**
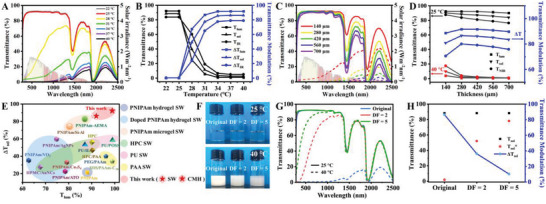
A) Transmittance spectra of CMH SW with interlayer thickness of 280 µm at different temperatures. The inset was the solar irradiance spectrum. B) The light transmittances (*T*_lum_, *T*_NIR_, and *T*_sol_) and light transmittance modulations (Δ*T*_lum_, Δ*T*_NIR_, and Δ*T*_sol_) at different temperatures. C) Transmittance spectra of CMH SWs with different interlayer thicknesses at 25 and 40 °C. D) The light transmittances (*T*_lum_, *T*_NIR_, and *T*_sol_) and light transmittance modulations (Δ*T*_lum_, Δ*T*_NIR_, and Δ*T*_sol_) in response to the increased interlayer thickness. E) Comparison of light management performances (*T*_lum_ at RT and Δ*T*_sol_ after phase transition) between our work with previously reported thermochromic smart windows.^[^ ^7^, ^12^, ^14^, ^15^, ^16^, ^18^, ^28^ ^]^ F) The photograph of as‐prepared and diluted CMH suspensions at 25 and 40 °C. G) Transmittance spectra of as‐prepared and diluted CMH suspensions, and H) the corresponding solar transmittances and transmittance modulations.

To systematically study the transmittance variation in response to the elevated temperature, UV–Vis‐NIR spectrophotometer was conducted every three degrees from 22 to 40 °C, without using a reference sample (**Figure** **3**A). The detailed luminous (*T*_lum_), NIR (*T*_NIR_), and solar (*T*_sol_) transmittances and the corresponding transmittance modulations (Δ*T*_lum_, Δ*T*_NIR_, and Δ*T*_sol_) were illustrated in Figure 3B. With the increase in temperature, the light transmittance reduced obviously. As the temperature rose from 25 to 28 °C, the luminous transmittance sharply decreased from 91.5% to 24.8%, while the NIR (780–2500 nm) transmittance decreased from 83.5% to 60.2%. The light blocking in visible light (380−780 nm) was attributed to the strong scattering behavior of the newly‐formed microgel particles (II) (20–200 nm). With the further size enlargement of microgel particles (II) (60–500 nm) at 31 °C, the produced scattering behavior led to obvious transmittance reduction in both visible and NIR regions. At 40 °C, the contractive microgel particles (I) merged microgel particles (II) into a broad size peak (100–600 nm) that can hinder the intense light radiation in full solar spectrum, leading to ultrahigh luminous (91.5%), NIR (78.7%) and solar (85.8%) transmittance modulations. Overall, the thermal‐induced ingenious size evolution of CMH suspension contributed to excellent broadband light management capacity.

In general, the solar modulation efficiency of thermochromic smart windows highly depended on the thickness, wherein the smart windows integrated thin thickness with remarkable solar regulation were preponderant for the industrialization of thermochromic energy‐saving windows. Here, five CMH SWs with different interlayer thicknesses (140, 280, 420, 560, and 700 µm) were fabricated to investigate their solar regulation capacity (Figure 3C,D). At 25 °C, all SWs exhibited ultrahigh luminous transmittance (>90.0%), which was favorable for perennial daylighting and heat gain in winter. As the temperature raised to 40 °C, the transmittance of all SWs decreased sharply in the full solar region. The Δ*T*_lum_ of five SWs were above 85.0%, illustrating their outstanding modulation capacity for visible light. With the increase of interlayer thickness, the NIR transmittance modulation augmented at the first stage, and then reduced slightly. The Δ*T*_NIR_ were 70.4%, 78.7%, 78.1%, 77.6%, and 75.7% for the SWs with interlayer thicknesses of 140, 280, 420, 560, and 700 µm, respectively, and the corresponding Δ*T*_sol_ achieved high values of 80.7%, 85.8%, 85.5%, 85.0%, and 83.3%, respectively. Overall, in the condition of 280 µm of interlayer thickness, the CMH SW displayed exceptional light management capacity with extraordinary luminous transmittance (*T*_lum_ = 91.5%) and solar transmittance modulation (Δ*T*_sol_ = 85.8%). According to the method reported in several literatures, using quartz glass as a reference sample to exclude the light occlusion by glass,^[^ ^7c,e^ ^]^ the interlayered CMH suspension exhibited overwhelming high luminous transmittance (99.0%) and solar modulation efficiency (93.0%) (Figure S16, Supporting Information). Impressively, the light management capacity in this work was much superior to most of the previously reported smart windows, such as PNIPAm‐based,^[^ ^12^, ^14^, ^15^, ^16^, ^18^, ^28^ ^]^ hydroxypropyl cellulose‐based^[^ ^28^, ^29^ ^]^ smart windows and so on^[^ ^7^, ^28^ ^]^ (Figure 3E).

The superior combination of thin interlayer thickness and eminent broadband light management capacity routed from the cooperative effect of ingenious size evolution and high concentration of as‐prepared CMH suspension. The thermal‐induced size evolution provided tunable scattering behaviors and dynamic spectral regulation capacity, while high concentration guaranteed the ultrahigh solar modulation efficiency. To confirm the important role of high concentration in the prominent light management capacity, the as‐prepared CMH suspension was diluted for investigating the variation of solar modulation. As illustrated in Figure 3F, the light blocking ability of diluted CMH suspensions at 40 °C was obviously reduced. Then, the corresponding CMH SWs were fabricated for quantifying the solar modulation capacity. Unless otherwise stated, the interlayer thickness was 280 µm in the following study. The spectral data clearly declared that the solar transmittance modulation (Δ*T*_sol_) attenuated sharply with the elevated dilution factor (DF) value. As the DF increased to 2 and 5, the Δ*T*_sol_ decreased to 36.5% and 10.7%, respectively, indicative of incompetence in blocking solar radiation.

**Figure 4:**
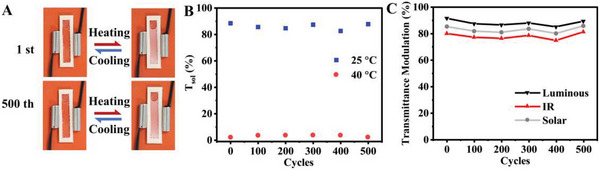
Remarkable cycling stability of CMH SW. A) The photographs of the first and 500th heating‐cooling cycles. B) Cycling solar transmittances before and after phase transition. C) The light transmittance modulations during 500 heating‐cooling cycles.

Long‐term stability is essential for the practical applications of energy‐saving windows. 500 continuous heating‐cooling cycles were performed on the CMH SW to investigate the cycling stability and durability in long‐term service. The photographs of first and 500th cycles visually demonstrated the repeatability of thermal‐induced solar transparent‐opaque conversion (**Figure** **4**A and Video S3, Supporting Information). As illustrated by the quantitative data in Figure 4B, during the 500 cycles, the CMH SW still maintained stable ultrahigh solar transmittance at 25 °C, together with ultrastrong solar modulation after phase transition. The transmittance modulations in luminous, NIR, and solar regions remained at significantly high levels, indicating excellent cycling stability and prominent durability (Figure 4C). Unlike previous PNIPAm‐based liquid smart windows requiring extra surfactant,^[^ ^8^, ^18^ ^]^ the as‐prepared CMH SW demonstrated inherent stability in multiple heating‐cooling cycles due to the strong electrostatic repulsion among microgel particles. Additionally, the CMH suspension was placed under a UV lamp (365 nm, 15 W) at close range to simulate long‐term sunlight exposure. As shown in Figure S17, Supporting Information, after 2 h of close UV irradiation, no precipitation or flocculation was produced in the CMH suspension, indicating significant UV stability. The distinct strategy to prepare high‐concentration and stable CMH suspension in this work also displayed a unique advantage in feasible fabrication for direct usage in real applications.

**Figure 5:**
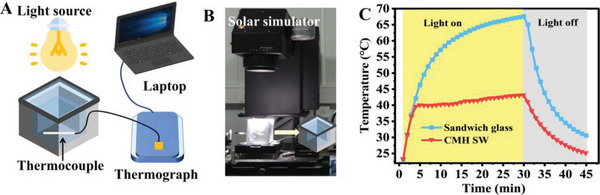
A) Schematic diagram and B) photograph of the simulation experiment device. C) Indoor temperature profiles of two model glass rooms under solar simulator exposure.

The remarkable advantages of feasible one‐step preparation, near‐room‐temperature transition, rapid response, ultrahigh *T*_lum_ and Δ*T*_sol_, and remarkable cycling stability made the CMH SW a desirable energy‐saving building material. Here, we quantified the indoor temperature management ability by monitoring the inner temperature of 70 × 70 × 70 mm^3^ glass room equipped with CMH SW, wherein the efficient area of SW was 50 × 50 mm^2^ (Figure S18, Supporting Information). As illustrated in **Figure** **5**A, the simulation experiment device consisted of light source, glass room, temperature acquisition, and recording system, wherein the metallic thermocouple was encased with a white paper for avoiding warming caused by simulated sunlight and acquiring accurate indoor temperature.^[^ ^17^ ^]^ A solar simulator, calibrated to 1.0 sun and air mass 1.5 illumination, acted as light source on the top of glass room (Figure 5B). After 30 min of solar irradiation, the indoor temperature of control glass room rapidly increased to 67.5 °C (Figure 5C). However, the phase transition of CMH SW was promptly triggered and completed after 5 min of illumination, resulting in dramatic reduction of inputted solar energy and plunge of heating rate of indoor temperature. After the complete phase transition, the indoor temperature raised merely 3.0 °C via heat conduction and damped solar irradiation, and stabilized at 43.0 °C at 30 min. The temperature difference between two glass rooms achieved an impressive value of 24.5 °C, meaning remarkable energy conservation capacity in summer (Figure S19, Supporting Information).

**Figure 6:**
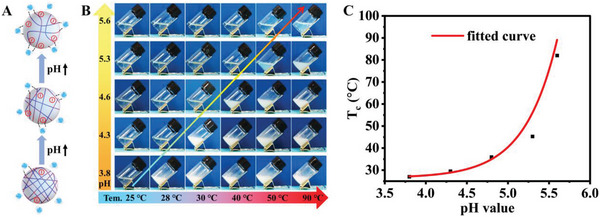
A) Schematic diagram of pH‐dependent structural evolution of P(NIPAm‐*co*‐AA) network. B) Transparency evolution of CMH suspensions with various pH values in response to raised temperature. C) The variation curve of transition temperature with the increased pH value.

Excitingly, the incorporation of hydrophilic and electronegative AA empowered the CMH with tunable transition temperature. Simple changing the pH value of CMH suspension can adjust the ionization of carboxyl groups and hydration degree of CMH, together with the intermolecular interaction (electrostatic repulsion and hydrogen bonding) and the interaction between CMH with water molecules, achieving flexible regulation on transition temperature.^[^ ^30^ ^]^ The pH value of the as‐prepared CMH suspension was 3.8 and displayed optimized transition temperature of 26.7 °C (Figure S12, Supporting Information). As the pH value of CMH suspension increased, much more ionized carboxyl groups enhanced the internal electrostatic repulsion as well as the hydrogen bonding between CMH with water (**Figure** **6**A), inducing obvious increment of transition temperature. As shown in Figure 6B, the transition temperature of CMH suspensions can be readily regulated by adjusting the pH values by adding low‐concentration NaOH solution (1.2 wt%). As determined by dynamic transmittance measurment, the transition temperatures of the CMH suspensions with pH values of 3.8, 4.3, 4.6, 5.3, and 5.6 were 26.9, 29.5, 35.9, 45.3, and 82.0 °C, respectively (Figure 6C and Figure S20, Supporting Information). Therefore, combined with the DSC data, we can conclude that the tunable transition temperatures of the CHM suspensions were in a broad range of 26.7 to 82.0 °C. The variation curve of transition temperature in response to increased pH value (Figure 6C) and amount of NaOH solution (Figure S21, Supporting Information) clearly illustrated the facile and feasible modulation on the transition temperature of CMH SW, benefiting to meet the diversified demands in various applications, such as body temperature monitoring. Overall, compared to other previously reported literatures, the strategy of copolymerization of NIPAm and AA via RATRP method to construct thermochromic CMH smart windows revealed terrific advantages.^[^ ^7^, ^12^, ^18^, ^28^, ^29^, ^31^ ^]^ As detailed illustrated in **Table** **1**, the CMH SW discovered appreciative transition temperature, rapid response, and recovery, prominent broadband light management in the condition of thin microgel layer, accompanied by broad‐range tunable transition temperature.

**Table 1:** Comparison of thermochromic performance between various hydrogel or microgel smart windows and this work

Sample	*T*_c_ [°C]	Response time [s]	Recovery time [s]	Thickness [µm]	*T*_lum_ [%]	Δ*T*_sol_ [%]	Regulatory media	*T*_c_ range [°C]	Versatility	Ref.
PNIPAm^a)^	32.0	–	–	78	85.8	49.6	–	–	×	[12b]
PNIPAm/Si‐Al hybrid^a)^	32.5	18–36	–	400	80.0	73.5	–	–	×	[28f]
HPMC^a)^	40.0	240	300–600	500	74.3	18.7	–	–	×	[31]
HPC/NaCl^a)^	38.0	–	–	350	86.1	25.7	NaCl	30–50	×	[29]
PU/POSS^a)^	30.0	–	–	200	99.0	58.2	POSS	18–80	×	[7b]
PNIPAm‐AEMA^b)^	31.0	300	–	240	87.2	81.3	–	–	×	[18]
PNIPAm/VO_2_@SiO_2_ ^b)^	32.0	150	–	3000	80.0	62.7	–	–	×	[19]
HPC/PAA^b)^	26.5	84	180	500	90.1	47.5	pH	10–45	×	[28e]
CMH SW^c)^	26.7	5	5	280	91.5	85.8	pH	27–82	√	
CMH suspension^c)^	26.7	5	5	280	99.0	93.0	pH	27–82	√	

^a)^ Hydrogel swart windows

^b)^ Microgel smart windows

^c)^ This work.

Note: “–” indicates not shown in the references.

**Figure 7:**
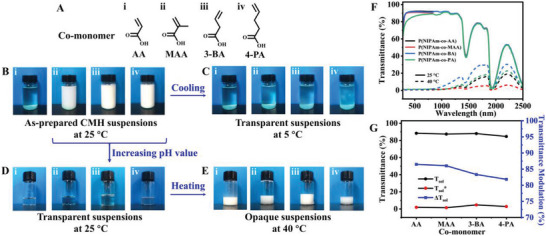
A) The chemical structures of various co‐monomers. The as‐prepared CMH suspensions at B) 25 and C) 5 °C. D) Solar transparent state and E) solar opaque state of various CMH suspensions at different temperatures. F) Transmittance spectra of various P(NIPAm‐*co*‐OA)‐based CMH suspensions at 25 and 40 °C, and G) the corresponding solar transmittances (*T*_sol_) and solar transmittance modulations (Δ*T*_sol_).

What's more, the proposed fabrication strategy can be generalized to different olefine acids (OA) as co‐monomers for constructing high‐performance thermochromic smart windows. In this work, methacrylic acid (MAA), 3‐butenoic acid (3‐BA), and 4‐pentenoic acid (4‐PA) were utilized as co‐monomers to prepare P(NIPAm‐*co*‐OA)‐based CMH suspensions (**Figure** **7**A and Table S2, Supporting Information). Due to that the hydrophobic long carbon chain or methyl group of the co‐monomers enhanced the internal interaction of CMH,^[^ ^32^ ^]^ the as‐prepared P(NIPAm‐*co*‐MAA)‐, P(NIPAm‐*co*‐BA)‐, and P(NIPAm‐*co*‐PA)‐based CMH suspensions demonstrated lower transition temperatures. As illustrated in Figure 7B,C, these CMH suspensions were opaque at RT, and tuned into highly transparent after cooling at 5 °C. Based on the flexible modulation of transition temperature, appropriate transition temperatures were obtained by simple adjusting the pH value of the CMH suspensions, realizing superior transparency at RT and high opaqueness at 40 °C (Figure 7D,E). As evaluated by UV–Vis‐NIR spectra, the modified P(NIPAm‐*co*‐MAA)‐, P(NIPAm‐*co*‐BA)‐, and P(NIPAm‐*co*‐PA)‐based CMH SWs revealed high luminous transmittance (>87%) and remarkable solar modulation efficiencies (86.3%, 83.3%, and 81.8%, respectively), manifesting the eminent light management capacity. Consequently, owing to the fascinatingly versatile strategy and extraordinary solar modulation capacity, the P(NIPAm‐*co*‐OA)‐based CMH SWs possessed great potential in commercial energy‐saving building materials.

## 3 Conclusion

In summary, we proposed a facile and feasible one‐step strategy to prepare P(NIPAm‐*co*‐AA)‐based CMH for liquid thermochromic smart windows by copolymerization of NIPAm and AA using RATRP method. The CMH suspension exhibited near‐room‐temperature transition (*T*_c_ = 26.7 °C), rapid response (5 s), tunable scattering behaviors, and broadband spectral regulation capacity resulting from the synergetic mechanism of thermal‐induced dissolution and aggregation of polymer chains with water capture and release of microgel particles. Without any post‐treatment, the as‐prepared high‐concentration CMH suspension was directly injected into a sandwich glass to construct energy‐saving windows, which allowed remarkable conversion from ultrahigh luminous transmittance (91.5%) at RT and extraordinary solar modulation efficiency (85.8%) after complete phase transition, achieving dramatic decline of indoor temperature of 24.5 °C. Notably, the transition temperature can be adjusted in a broad range of 26.7–82.0 °C by simply varying the pH value of CMH suspension. More meaningfully, the strategy can be generalized to various co‐monomers (AA, MA, 3‐BA, 4‐PA, etc.) for yielding diversified thermochromic energy‐saving smart windows with excellent light management capacity. The facile yet versatile strategy will open an avenue to construct commercial and scalable energy‐efficient smart windows for the goal of carbon emission reduction and carbon neutrality.

## 4 Experimental Section

### Materials

*N*‐isopropylacrylamide (NIPAm, monomer, 98%), acrylic acid (AA, monomer, 99%) *N*,*N*‐methylenebis(acrylamide) (MBA, crosslinker, 97%), potassium persulphate (KPS, initiator, 99%) were purchased from Alfa Aesar. Copper salts (CuCl_2_, CuSO_4_, and Cu_3_Cit_2_) were purchased from Sinopharm Chemical Reagent Co., Ltd. KPS was purified via recrystallization in DI water. Other reactants were used without further purification.

### Fabrication of CMH Suspension and Smart Window Device

The CMH suspension was synthesized via feasible one‐step method. Briefly, NIPAm (1.73 g), AA (206 µL), MBA (0.01 g, 0.35 mol% of monomer), KPS (0.025 g, 0.5 mol% of monomer), and copper citrate (0.02 g) were added into 18 mL of H_2_O to form a suspension with copper citrate nanoparticles. The mixture was polymerized at 70 °C for 3 h to obtain CMH suspension. The CMH SW was fabricated by pouring the as‐prepared CMH suspension into sandwich glass, wherein the interlayer thickness was 140, 280, 420, 560, and 700 µm. The as‐prepared CMH suspension (*w*_1_) was freeze‐dried, washed with hot water several times to remove the residual monomers, and then freeze‐dried again to obtain the mixture of dry linear P(NIPAm‐*co*‐AA) chains and Cu_3_Cit_2_@P(NIPAm‐*co*‐AA) core–shell microparticles (*w*_2_), and total mass fraction in the suspension was calculated by *w*_2_/*w*_1_ × 100%. The as‐prepared CMH suspension (*w*_1_) was centrifuged to obtain separated microparticles, which was further freeze‐dried to obtain the dry Cu_3_Cit_2_@P(NIPAm‐*co*‐AA) core–shell microparticles (*w*_3_) and corresponding mass fraction of core–shell microparticles (*w*_3_/*w*_1_ × 100%). The mass fraction of dry linear P(NIPAm‐*co*‐AA) chains was calculated by (*w*_2_/*w*_1_‐*w*_3_/*w*_1_) × 100%. The control P(NIPAm‐*co*‐AA) solution with identical concentration was prepared via the same method without crosslinker. The control Cu_3_Cit_2_@P(NIPAm‐*co*‐AA) core–shell microgel suspension with identical concentration was obtained via centrifugation and dispersion.

### Characterization

The morphologies of microgel particles at different temperatures were evaluated via the super depth of field microscope (Leica DM/LP) using vertical or lateral light sources. The refractive index was recorded via a refractometer (IR140, Insmark Instrument Technology Co., Ltd.) at the wavelength of 589.3 nm. DSC analysis was conducted on TA Q2000 over the temperature range of 20–40 °C with a heating/cooling rate of 2 °C min^−1^ under nitrogen flow, and the endothermic peak temperature referred to as the transition temperature. A light transmittance meter (LH‐221, Lianhuicheng Technology Co., Ltd.) was also utilized to measure the transition temperature in the wavelength of 380–780 nm with a heating rate of 2 °C min^−1^, and the temperature corresponding to transmittance of 50% was noted as the transition temperature.^[^ ^28e^ ^]^

The transmittance spectra were obtained using a UV–Vis‐NIR spectrophotometer (Cary 5000, Agilent, USA) at normal incidence, without using a reference sample. The luminous transmittance *T*_lum_ (380–780 nm), NIR transmittance *T*_NIR_ (780–2500 nm), and solar transmittance *T*_sol_ (280–2500 nm) can be calculated by Equation (1).^[^ ^18^ ^]^

(1) $$T_{\mathrm{sol}/\mathrm{lum}/\mathrm{NIR}}\;=\int \varphi_{\mathrm{sol}/\mathrm{lum}/\mathrm{NIR}}\left(\lambda \right)T\left(\lambda \right)d\lambda /\int \varphi_{\mathrm{sol}/\mathrm{lum}/\mathrm{NIR}}\left(\lambda \right)d\lambda$$

where *T*(*λ*) denotes the recorded transmittance at a particular wavelength, *φ*_lum_ is the standard luminous efficiency function for the photonic vision of human eyes, and *φ*_sol/NIR_ is the solar/NIR irradiance spectrum for air mass 1.5.

The corresponding transmittance modulations can be calculated by Equation (2).

(2) $$\Delta T_{\mathrm{sol}/\mathrm{lum}/\mathrm{NIR}}\;=\;T_{\mathrm{sol}/\mathrm{lum}/\mathrm{NIR}}\;(\leq T_{c})\;-\;T_{\mathrm{sol}/\mathrm{lum}/\mathrm{NIR}}\;(\geq T_{c})$$

From the measured transmittance spectra, the light transmittances (*T*_lum_, *T*_NIR_, and *T*_sol_) at 25 and 40 °C can be obtained via Equation (1). Then, the light transmittance modulations (Δ*T*_lum_, Δ*T*_NIR_, and Δ*T*_sol_) were calculated via Equation (2). Δ*T*_sol/lum/NIR_ = *T*_sol/lum/NIR_ (25 ^○^C) − *T*_sol/lum/NIR_ (40 ^○^C).

Dynamic light scattering measurement and zeta potential were performed on the Marvern Zetasizer nano ZS 90 (Georgia Tech, UK) with a laser at the wavelength of 633 nm. The zeta potentials (*ξ*) were obtained from Equation (3).^[^ ^27^ ^]^

(3) $$\xi \;=\frac{\mu U_{e}}{\varepsilon }$$

where *U*_e_ (m^2^ s^−1^ V^−1^) is the electrophoretic mobility, *ε* (F m^−1^) is the electric permittivity, and *µ* (Pa s) is the dynamic viscosity of the liquid.

### Long‐Term Cycling Stability Measurement

The 500 continuous heating‐cooling cycles were performed via a combined device with a heating panel and a thermoelectric cooler, accompanied by a temperature control system (PY‐SM5, PinYi Electrical Appliance Co., Ltd.). During the heating process from 25 to 40 °C, only the heating panel worked. Likewise, during the cooling process from 40 to 25 °C, only the thermoelectric cooler worked. It took 80 s to accomplish a heating‐cooling cycle.

### UV Stability Measurement

The CMH suspension was placed under a UV lamp (365 nm, 15 W) at close range for 2 h to observe whether there was precipitation or flocculation formation.

### Indoor Temperature Regulation Capability Measurement

The simulation experiment device consisted of a light source, glass room, temperature acquisition, and recording system. The experimental and control glass rooms (70 × 70 × 70 mm^3^) were respectively equipped with CMH SW and sandwich glass (efficient area: 50 × 50 mm^2^) as top, and insulated silica aerogel (thickness: 10 mm) to avoid indoor energy loss. The metallic thermocouple was encased with a white paper for avoiding warming caused by simulated sunlight and acquiring accurate indoor temperature.^[^ ^17^ ^]^ A solar simulator (Newport 94023A, USA), calibrated to 1.0 sun (100 mW cm^−2^) and air mass 1.5 illumination, acted as a light source on the top of the glass room. The real‐time indoor temperatures of two glass rooms were recorded during the whole experimental process. The indoor temperature regulation capability can be evaluated by the temperature difference of two model glass rooms.^[^ ^18^ ^]^

## Conflict of Interest

The authors declare no conflict of interest.

## Supporting information

Supporting Information

Supplemental Video 1

Supplemental Video 2

Supplemental Video 3

## Data Availability

Research data are not shared.
