# Supplementary material for: A Facile yet Versatile Strategy to Construct Liquid Hybrid Energy‐Saving Windows for Strong Solar Modulation
Source: Adv Sci (Weinh). 2023 Jan 20;10(10):2206044. doi: 10.1002/advs.202206044 (PMC10074053; doi:10.1002/advs.202206044)
Supplement: Supplementary file 1 — Supporting Information [file ADVS-10-2206044-s001.pdf]

ADVANCED SCIENCE
Open Access

Supporting Information

for *Adv. Sci.*, DOI 10.1002/advs.202206044

A Facile yet Versatile Strategy to Construct Liquid Hybrid Energy-Saving Windows for Strong Solar Modulation

Jichang Li, Pengyu Gu, Hongyu Pan, Zhiyuan Qiao, Jianfeng Wang, Yanxia Cao, Wanjie Wang and Yanyu Yang**

Supporting Information

A Facile Yet Versatile Strategy to Construct Liquid Hybrid Energy-Saving Windows for Strong Solar Modulation

Jichang Li, Pengyu Gu, Hongyu Pan, Zhiyuan Qiao, Jianfeng Wang, Yanxia Cao, Wanjie Wang and Yanyu Yang**

Part 1. Figures

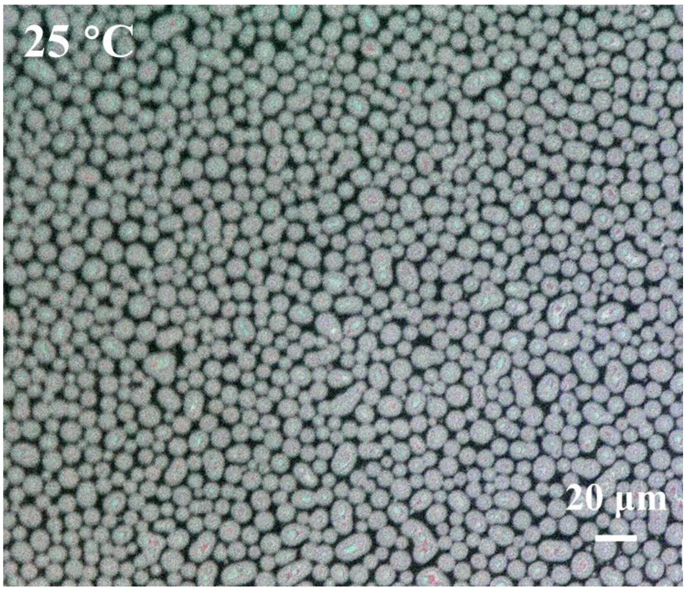

Figure S1. The core-shell microgel particles (I) observed by SDFM using lateral light source.

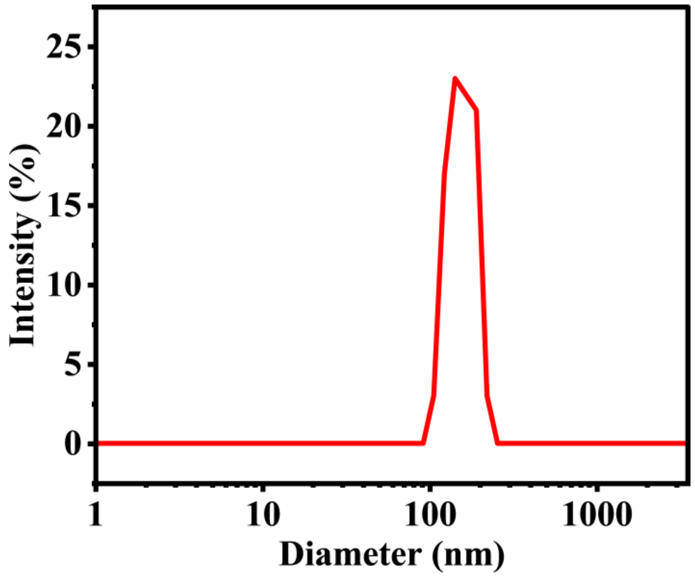

Figure S2. Particle size distribution of Cu_3Cit_2 nanoparticles.

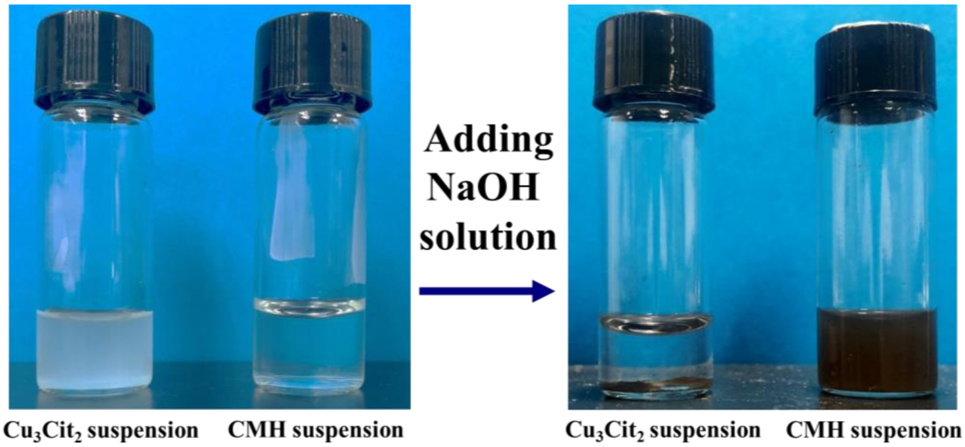

Figure S3. Photographs of (left) Cu_3Cit_2 and (right) CMH suspension before and after adding equal NaOH solution.

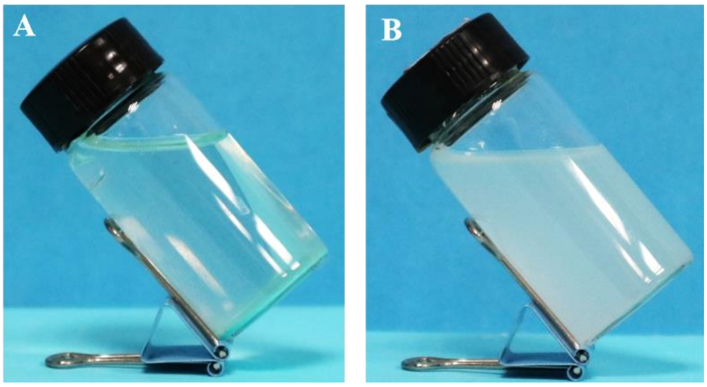

Figure S4. Photographs of A) transparent CMH suspension (6.8 wt%) and B) semitransparent PNIPAm microgel suspension (1 wt%) at RT.

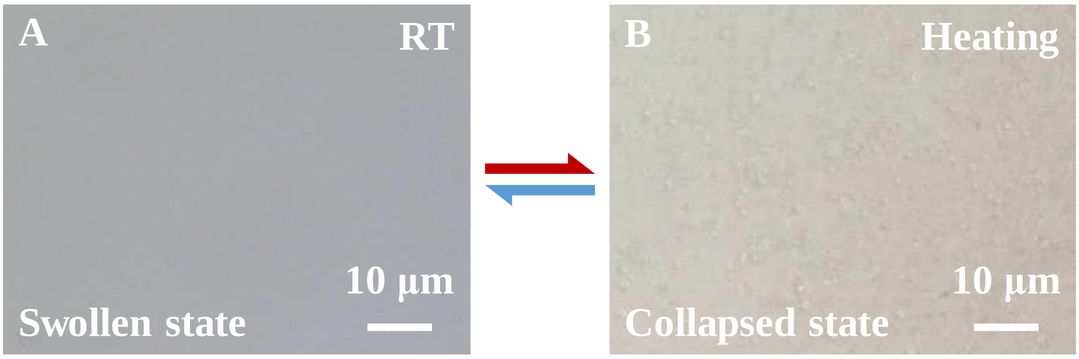

Figure S5. The thermal-induced phase transition of CMH suspension observed by SDFM using vertical light source: A) swollen state at RT, B) collapsed state upon heating. Detailed information shown in Video S1.

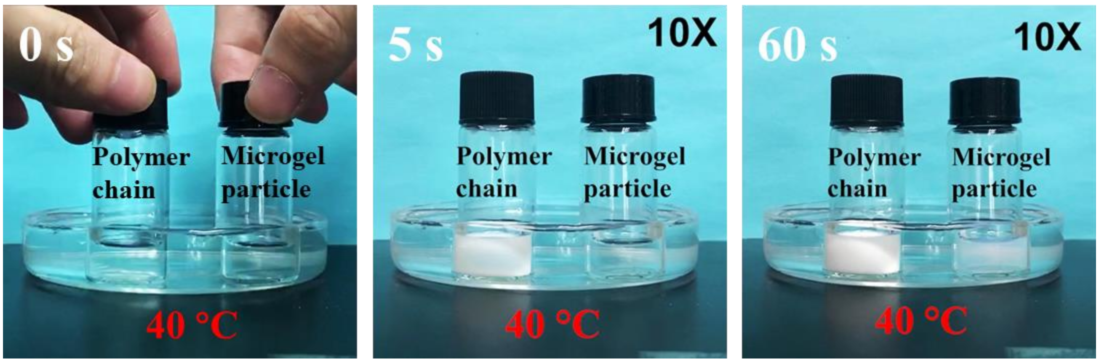

Figure S6. Photographs of the separated P(NIPAm-co-AA) solution and separated Cu_3Cit_2@P(NIPAm-co-AA) core-shell microgel suspension in hot water of 40 ^oC.

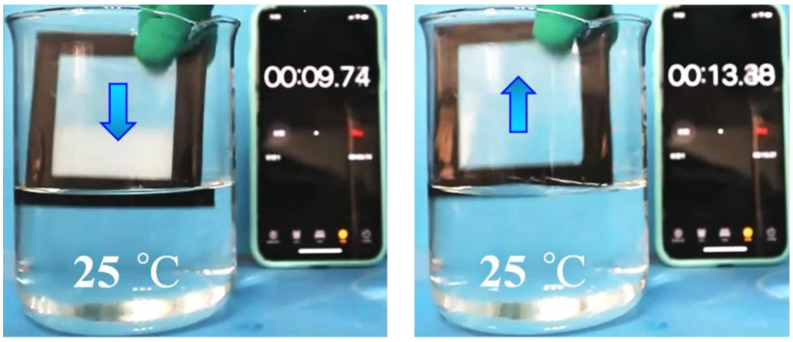

Figure S7. Fast recovery of CMH SW. Detailed information shown in Video S2.

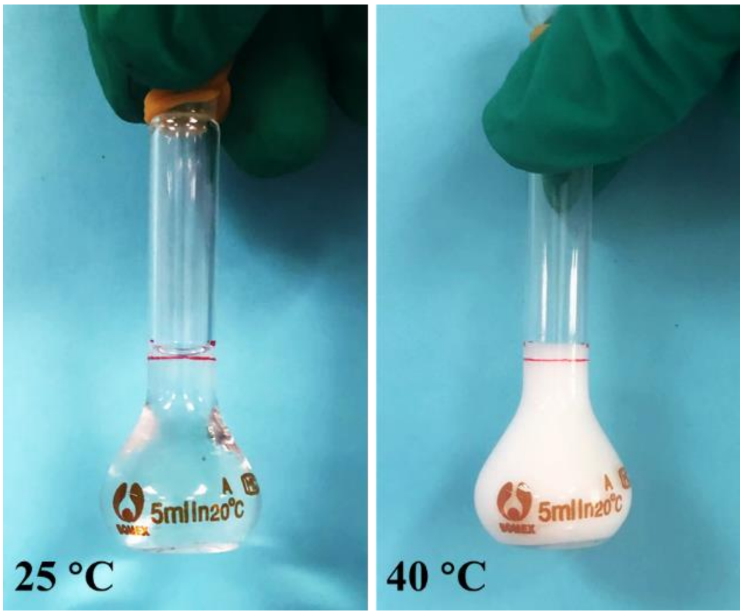

Figure S8. Photographs of the volumetric flask filled with CMH suspension at (left) 25 and (right) 40 °C.

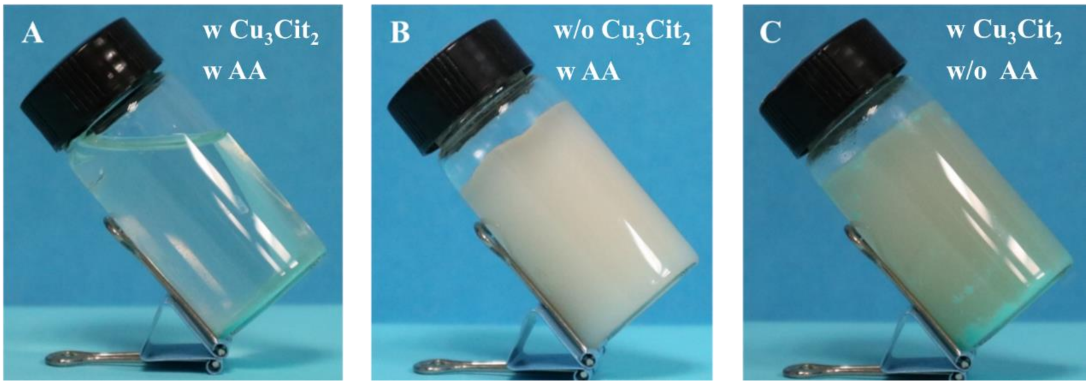

Figure S9. Photographs of A) CMH suspension with Cu_3Cit_2 salt and AA, B) P(NIPAm-co-AA) bulk hydrogel without Cu_3Cit_2 salt, and C) PNIPAm bulk hydrogel without AA.

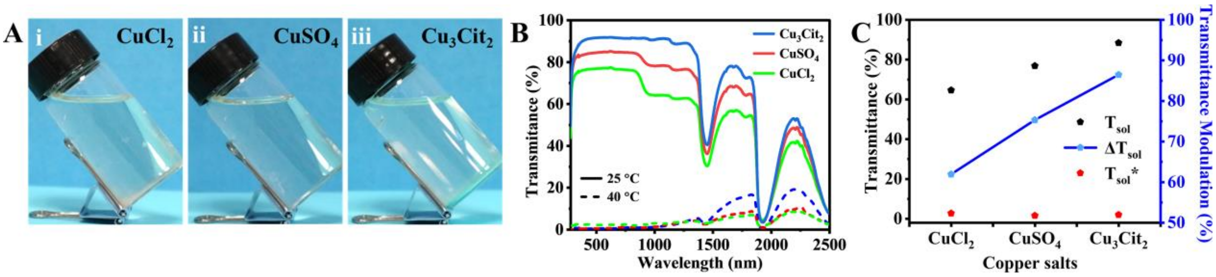

Figure S10. A) CMH suspensions prepared with different copper salts. B) Transmittance spectra of three CMH suspensions at 25 and 40 °C, and C) their corresponding solar transmittances (T_{sol}) and solar transmittance modulations (ΔT_{sol}). The interlayer thickness was 280 μm.

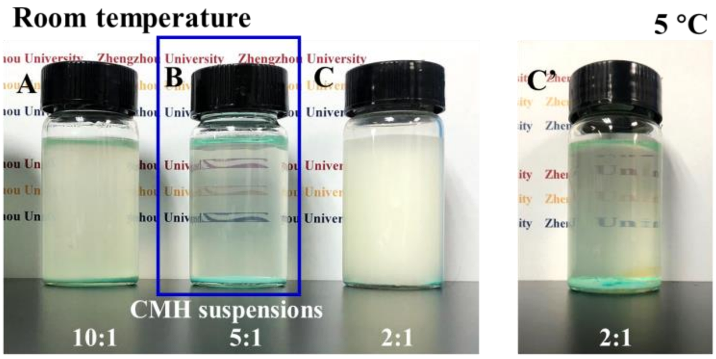

Figure S11. Photographs of A-C) CMH suspensions with various ratios of $n_{(NIPAm)}$:$n_{(AA)}$. C') Transparent CMH suspension ($n_{(NIPAm)}$:$n_{(AA)}$ = 2:1) at 5 °C.

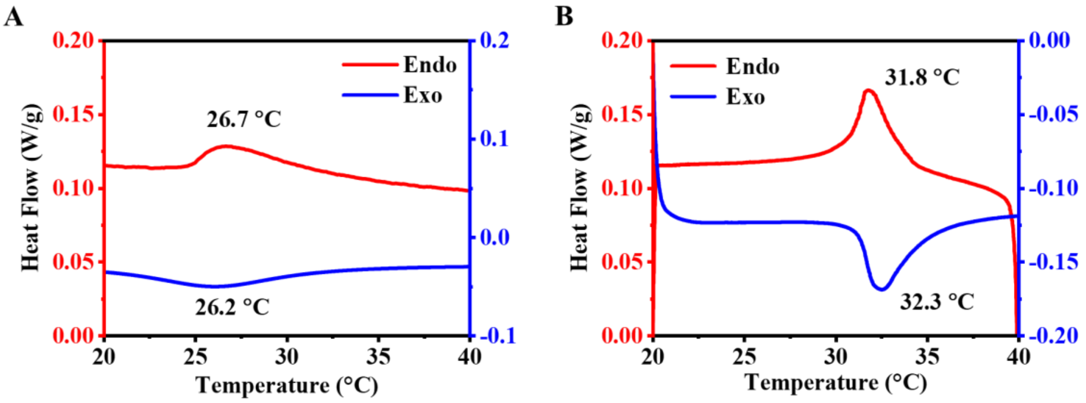

Figure S12. DSC curves of A) CMH suspension with $n_{(NIPAm)}$:$n_{(AA)}$ of 5:1 and B) pure PNIPAm hydrogel in the endothermic (Endo) and exothermic (Exo) process.

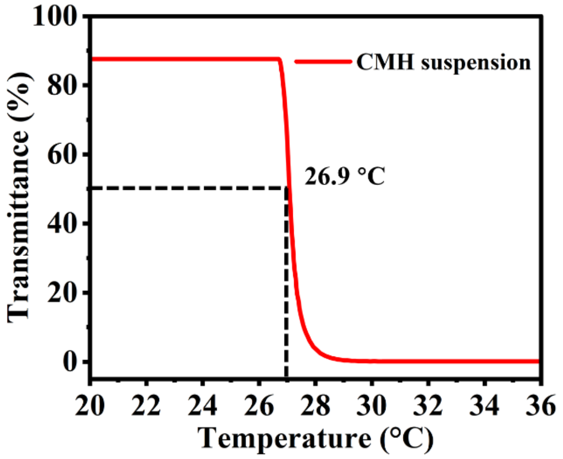

Figure S13. Dynamic transmittance of CMH suspension in a quartz cuvette (thickness: 1 cm) during heating process.

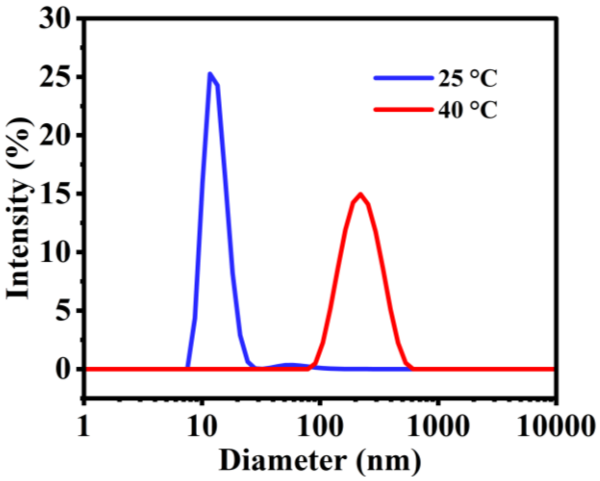

Figure S14. DLS curves of the separated P(NIPAm-co-AA) solution at 25 and 40 ^{o}C.

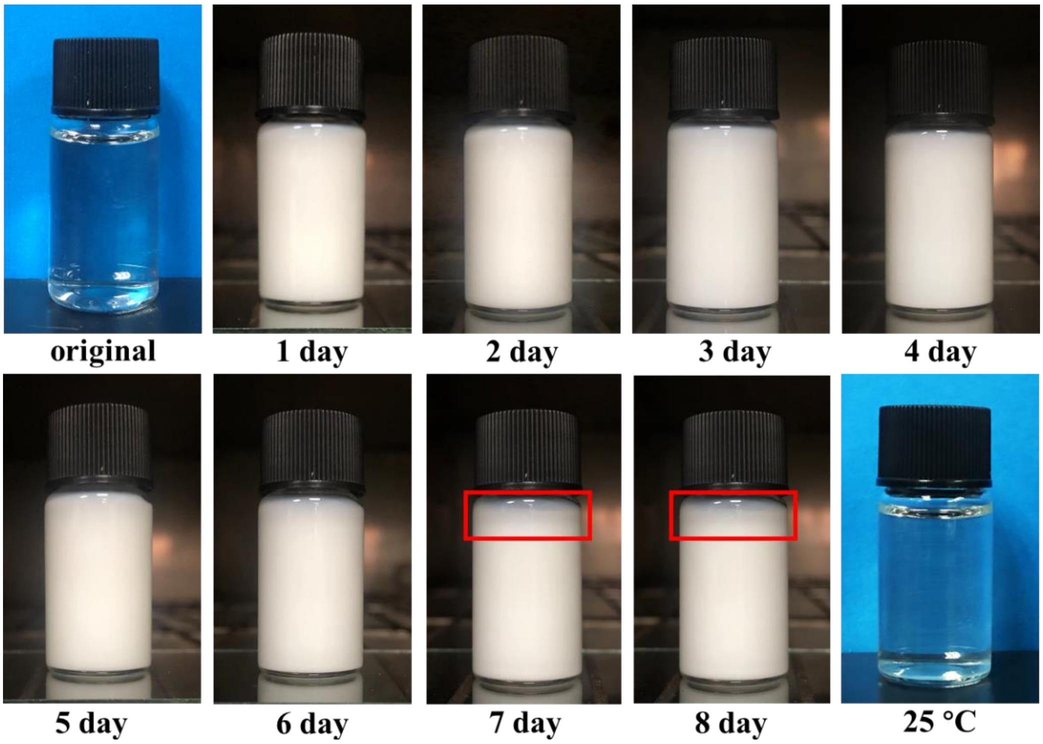

Figure S15. Photographs of CMH suspension in an oven at 45 °C for several days.

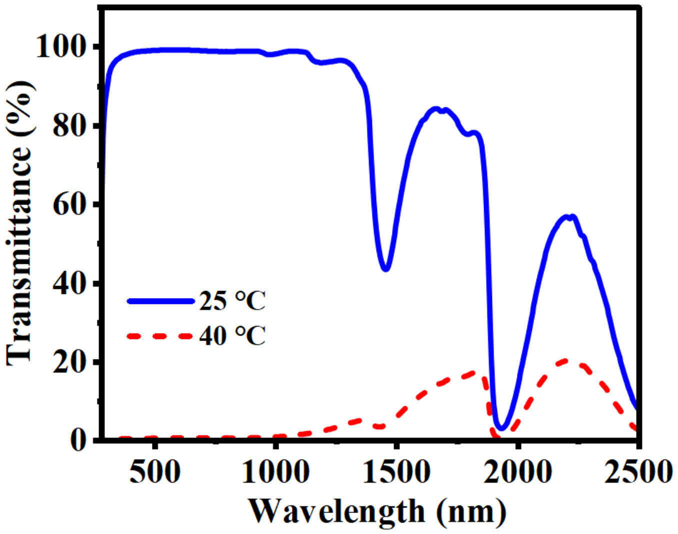

Figure S16. Transmittance spectra of CMH suspension with thickness of 280 μm, using quartz glasss as a reference sample.

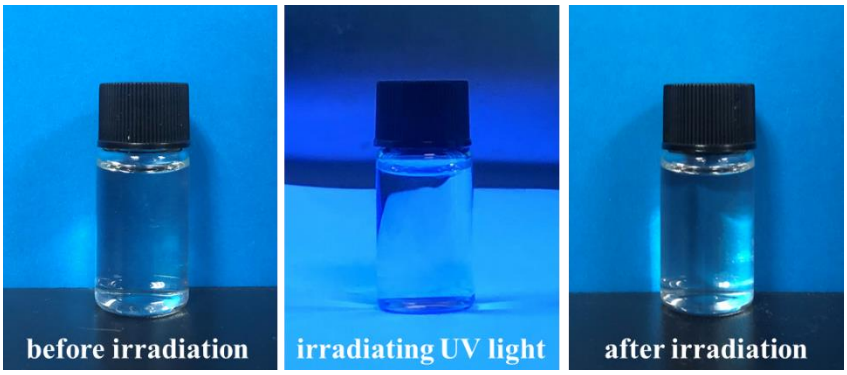

Figure S17. Photograph of CMH suspension (left) before, (middle) during and (right) after UV irradiation.

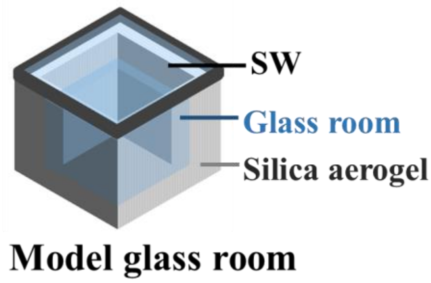

Figure S18. Schematic diagram of the model glass room.

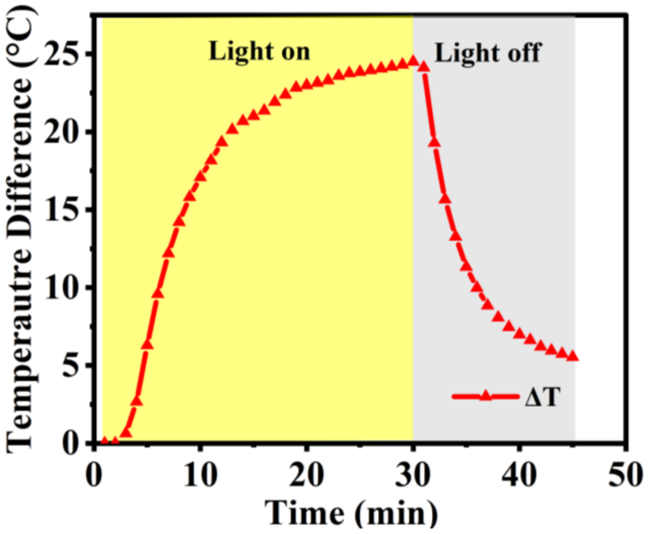

Figure S19. Time-dependent temperature difference between two model glass rooms.

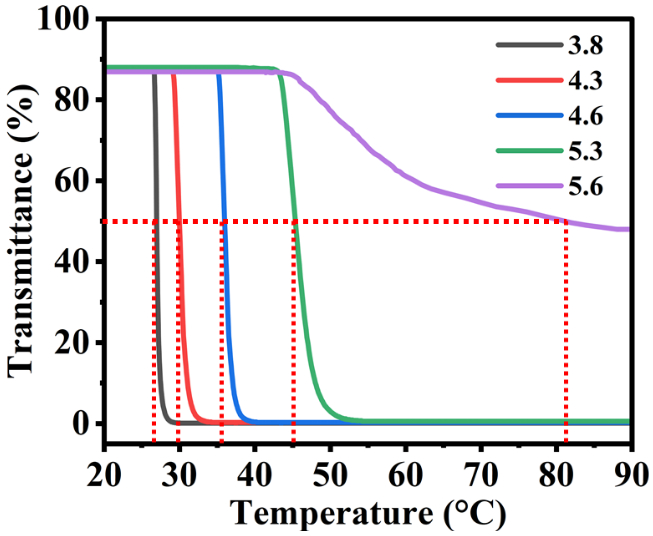

Figure S20. Dynamic transmittance of CMH suspensions with various pH values during heating process.

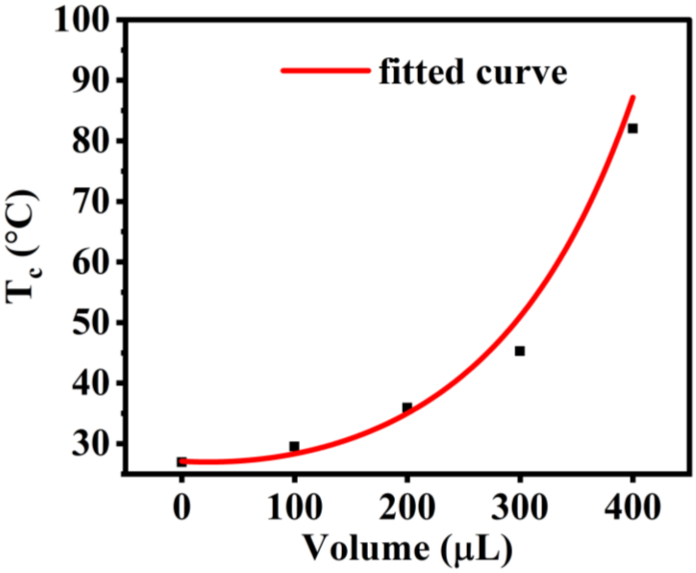

Figure S21. The variation curve of transition temperature with additive amount of NaOH solution (1.2 wt%).

Part 2. Tables

Table S1. Feed composition of CMH suspensions with various molar ratio of monomers.

Sample	V(NIPAm)/mL[a)]	V(AA)/mL[a)]	$n_{(NIPAm)}$:$n_{(AA)}$[a)]	m(KPS)/g[b)]	m(MBA)/g[c)]	m(Cu_3CA_2)/g
1	15	3	5:1	0.025	0.01	0.02
2	16.5	1.5	10:1	0.025	0.01	0.02
3	12	6	2:1	0.025	0.01	0.02

[a)]The concentration of NIPAm and AA solutions were 1 mol/L, and the volume ratio was the molar ratio; [b)]The content of KPS was 0.5 mol% of monomers; [c)]The content of MBA was 0.35 mol% of monomers.

Table S2. Feed composition of various CMH suspensions with different co-monomers.

Sample	Co-monomer	V(NIPAm) /mL	V(co-monmmer) /mL	$n_{(NIPAm)}$: $n_{(co\text{-}monomer)}$[a)]	m(KPS) /g[b)]	m(MBA) /g[c)]	m(Cu_3CA_2) /g
i	AA	15	3	5:1	0.025	0.01	0.02
ii	MAA	15	3	5:1	0.025	0.01	0.02
iii	3-BA	12	6	2:1	0.025	0.01	0.02
iv	4-PA	12	6	2:1	0.025	0.01	0.02

[a)]The concentration of NIPAm and AA solutions were 1 mol/L, and the volume ratio was the molar ratio; [b)]The content of KPS was 0.5 mol% of monomers; [c)]The content of MBA was 0.35 mol% of monomers. As for 3-BA, 4-PA, the polymerization systems with $n_{(NIPAm)}$:$n_{(co\text{-}monmmer)}$ = 5:1 generated bulk hydrogel, whereas the polymerization systems with $n_{(NIPAm)}$:$n_{(co\text{-}monmmer)}$ = 2:1 produced CMH suspensions.
